# An Approach to the Design of a Particulate System for Oral Protein Delivery .II. Preparation and Stability Study of rhGH-Loaded Microspheres in Simulated Gastrointestinal Fluids

**Published:** 2011

**Authors:** Nastaran Nafissi Varcheh, Reza Aboofazeli

**Affiliations:** a *Department of Pharmaceutical Biotechnology, School of Pharmacy, Shaheed Beheshti University of Medical Sciences, Tehran, Iran.*; b *Department of Pharmaceutics, School of Pharmacy, Shaheed Beheshti University of Medical Sciences, Tehran, Iran.*

**Keywords:** Microencapsulation, Oral protein delivery, Biodegradable polymer, Microparticle, Resomer^®^

## Abstract

The delivery of therapeutic proteins has gained momentum with development of biotechnology. However, large molecular weight, hydrophilic nature and susceptibility to harsh environment of gastrointestinal tract (GIT) resulted in low absorption. The main objective of this work was the design of a particulate system for oral delivery of recombinant human growth hormone (rhGH) on the basis of particle uptake mechanism in GIT. Biodegradable protein-loaded microspheres were prepared using Resomers (RG207, RG756 and RG505) by double emulsion methods. Aqueous solution of protein and freshly prepared rhGH-zinc complex were used for loading process. Various analytical methods, including fluorescence spectroscopy, SDS-PAGE electrophoresis and reversed-phase chromatography, were set up for the quantification and qualification of rhGH before and after the formulation and fabrication procedures. At the optimum conditions, microspheres were mostly below 10 μm with relatively high protein loading (> 50%). Obtained data showed that the stability of protein did not change during the formulation and microencapsulation processes. Results also showed that the encapsulation process in the presence of zinc caused no detectable change in the protein chemical stability. *In-vitro *stability study of microspheres in different simulated GI media indicated that the entrapped protein was physically stable. Less than 20% of rhGH was released from the microspheres incubated in both simulated stomach and intestine fluids for 3 and 6 h, respectively.

## Introduction

Proteins and peptides are potent molecules and used for the treatment of various chronic and life-threatening diseases. Although recent advances in biotechnology have led to the production of various proteins, their administration for therapeutic purposes has been limited exclusively to injectable routes, due to their short half-lives, instability in the GI tract and incapability of diffusing through biological membranes (1, 2). However, because of the need for frequent injections and discomfort of this invasive route, tremendous efforts have also been made to the development of oral delivery of these macromolecules (3, 4).

In so doing, one of the key requirements is the maintenance of protein integrity during its stay in the stomach and intestines. Among the innovative approaches employed, biodegradable polymeric microspheres prepared in particular with poly (lactic-co-glycolic acid) (PLGA) have been extensively investigated for the protection and delivery of the intact protein drugs. In this regard, various proteins have been microencapsulated within PLGA microspheres (5-14)

Recombinant human growth hormone (rhGH) has been approved for the treatment of pediatric hypopituitary dwarfism and adults with growth hormone deficiency (15). This protein is a single-chain peptide composed of 191 amino acids and two disulfide bridges with a molecular mass of approximately 22000 daltons. Like other protein drugs, it is susceptible to various chemical and physical instability reactions. Dosing regimens of rhGH usually include a few subcutaneous injections daily or per week which may continue for several years (16).

In our previous study, *in-vitro *stability of microspheres prepared by a series of commercially available poly (lactic acid) (PLA) and poly (lactic-co-glycolic acid) (PLGA) copolymers were monitored and compared in different simulated GI fluids (17). In the current research, rhGH-loaded microspheres were fabricated and protein stability and *in-vitro *polymer degradation/erosion behaviors for PLA and PLGA microspheres were investigated. In this regard, microspheres prepared based on the double emulsion technique were incubated in various GI simulated fluids and their integrity and morphology were then examined. Physical and chemical stability of protein before and after microencapsulation and following the incubation of microspheres in simulated GI fluids were also evaluated.

Although a sustained release dosage of rhGH as an injectable suspension of PLGA microspheres for subcutaneous administration has been developed successfully (27), it was hypothesized that by choosing an appropriate PLA/PLGA polymer that degrades very slowly for a desired period in simulated GI fluids, rhGH-loaded microparticles, as a desired particulate system for oral delivery of rhGH, could be achieved.

## Experimental

*Materials*

Resomer^®^ RG505 (PLGA, dl-LA:GA 50:50, I.V. = 0.7 dL/g), Resomer^®^ RG756 (PLGA, dl-LA:GA 75:25, I.V. = 0.8 dL/g) and Resomer^®^ R207 (PLA, poly dl-LA, I.V. = 1.5 dL/g) were purchased from Boehringer Ingelheim Co. (Germany). Polyvinyl alcohol (Mowiol^®^, 8-88) was a gift from Kuray Co. (Germany). rhGH was obtained from Novo Nordisk Pharmaceutical Co. (Denmark). Somatropin coded NIBSC- 98/574 (Hertfordshire, UK), accepted by the Expert Committee on Biological Standardization of WHO in 2001 was applied as the standard of rhGH. Physiogel^®^ (succinylated gelatin) 4% was supplied from B. Braun Medical AG (Emmenbrucke, Switzerland). Pepsin, pancreatin, TEMED (N, N, N′, N′-Tetramethylethylenediamine) and Coomassie Brilliant Blue R-250 (electrophoresis grade) were purchased from Sigma Co. (USA). Lecithin was provided from Lucas Meyer Co. (Germany). Low molecular weight marker kit for SDS-electrophoresis (LMW-SDS; contained 6 various polypeptide in the range of 14.4 to 97 KDa), lactate diagnostic kit and sodium taurocholate were obtained from Amersham Biosciences Co. (Sweden), Randox Laboratories (UK) and Difco Laboratories (USA), respectively. All other chemicals were purchased from Merck Co. (Germany). Purified water prepared by a Millipore system (Millipore Corp., USA) was used for all experiments.

*Methods*

*Preparation of rhGH-Zn complex*

rhGH-Zn precipitate was prepared according to a previously published method (18) with some modifications. Briefly, an aqueous protein solution (10 mg/mL) in 5 mM Tris buffer (pH = 8) containing 1% polyvinyl alcohol (PVA) was mixed with zinc chloride to reach the desired Zn: rhGH molar ratio. pH was then adjusted to 7.4 and the resultant suspension was incubated for 1 h while stirring magnetically at room temperature.

*Preparation of rhGH-loaded microspheres*

Biodegradable microspheres of rhGH were prepared by two methods. In method I, which was a conventional double emulsion technique, an aqueous solution of protein was applied. To this end, a solution of 10 mg/mL rhGH in 5 mM Tris buffer (pH = 8) containing 1% PVA was prepared. In method II, which was a modified technique similar to that applied in the previous study on lysozyme (19), a freshly prepared rhGH-zinc complex was used. Briefly, after dissolving 100 mg polymer in 2 mL dichloromethane, 200 μL of protein solution or suspension equivalent to 2 mg pure protein was added to this organic phase. 50 μL Physiogel® was also added to the aqueous phase for some batches. The obtained mixture was emulsified using a Diax 900 homogenizer (Heidolph Co., Germany) at 26000 rpm for 60 sec. This o/w emulsion was then transferred immediately into 60 mL cooled (15°C) aqueous PVA solution (5% w/v), while stirring at 1300 rpm for 2 min by a propeller stirrer (RZR50, Heidolph Co., Germany). The resultant double emulsion (w/o/w) was added within 2-3 min into 600 mL of 1% w/v pre-chilled (4°C) aqueous PVA solution and the mixture was magnetically shaken for 7 to 18 h at 300 rpm and 4-8°C. The prepared microspheres were harvested by filtration and washed with 500 mL of cold (4°C) distilled water. Final drying was performed at 4°C under reduced pressure within 24 h. 

*Morphological studies*

Light and scanning electron microscopy (SEM) were used for studying the size and morphological properties of protein-loaded microspheres. For SEM studies, the microspheres were mounted on aluminum stubs, vacuum-coated with a gold film using a sputter coater (SCDOOS, Bal-TEC Co., Switzerland) and directly analyzed by scanning electron microscope (XL30, Philips Co., Netherlands).

*Determination of protein loading*

Protein loading was determined by dividing the actual amount of rhGH in a pre-weighed sample of microspheres by its theoretical amount. In this regard, 20 mg of microspheres were dissolved in 1.5 mL 90% v/v glacial acetic acid. The protein was assayed spectrofluorimetrically using a Fluorimeter 6200 (Jenway Co., UK) at 295 nm excitation and 340 nm emission wavelengths. Dilution was performed by 90% v/v acetic acid, as necessary.

*Determination of chemical stability and aggregation of protein*

Sodium dodecyl sulfate-polyacrylamide gel electrophoresis (SDS-PAGE) was performed using a VEU-7709 vertical tank and EPS-7602 power supply (Payapajoohesh Co., Iran) and reversed-phase high performance liquid chromatography (RP-HPLC) was carried out using a computer-controlled Shimadzu HPLC system (Japan) equipped with two pumps (LC-10 ADvp), a system controller (SCL-10Avp), a degasser ((DGU-14A) and a diode array detector (SPD-M10vp). These techniques were applied for studying physical and chemical stability of rhGH before and after microencapsulation and following the incubation of microspheres in simulated GI fluids, respectively.

Electrophoresis analysis was done by dispersing 5 mg microspheres in the sample buffer, followed by incubation for 1 h at room temperature. The samples were then boiled for 10 min before loading in the wells. Chromatography analysis was performed by the method developed for the separation of deamidated and oxidized forms of hGH. Briefly, a polymeric poly (styrene-co-divinylbenzene) column (PRP-3, 150*4.1 mm, Hamilton Co., Switzerland) with a pore size of 300 Å and particle size of 10 μm was used and a linear gradient of 0.1% v/v TFA/acetonitrile and TFA/ water (pH = 2.00) mixture with a 1 mL/min flow rate was applied as the mobile phase for the identification of rhGH and detection of any degradation products at 215 nm. Extraction of the loaded protein was done by dissolving 50 mg microspheres in 4 mL of 90% acetic acid in order to obtain a clear solution. 4 mL of water was then added and shaking was gently continued to decrease the polymer solubility and facilitate the precipitate formation. After 15 min, the mixture was centrifuged (Harrier 18/80, Sanyo Co., Japan) at 4°C and 4500 rpm for 30 min and the supernatant was removed carefully and filtered under the nitrogen pressure using polysulfone membrane filter (Sartorius Co., Germany). The protein remained on the filter surface was washed by 2 mL of cold water and the resultant concentrated sample was injected to the reversed-phase chromatography column. The concentration of extracted rhGH sample was approximately 1 mg/mL and the injection volume was 20 μL.

*Determination of initial release of protein from microspheres*

In order to evaluate the potential of *in-vitro *protein release from microspheres within the first 24 h, a phosphate buffer USP (pH = 7.4) containing 0.9% sodium chloride was prepared. Pre-weighed microspheres (20 mg) were then transferred to 1.5-mL microcentrifuge tubes and incubated in 1 mL simulated medium at 37°C using an orbital incubator (SI 150, Stuart Scientific Co., UK), while shaking at 75 rpm. After centrifugation (Harrier 18/80, Sanyo Co., Japan) at 4°C and 4500 rpm for 30 min, the supernatants were analyzed spectrofluorimetrically. The percent of initial release of protein was calculated by dividing the released amount of protein by the whole content of loaded protein in microspheres.

*Incubation and stability assessment of microspheres in simulated GI fluids*

Similar to our previously reported study on the evaluation of *in-vitro *polymer stability in GI tract (17), four different media including SGF-a (USP XXVII simulated gastric fluid with pepsin; pH = 1.2), SIF-a (USP XXVII simulated intestinal fluid with pancreatin; pH = 6.8), FaSSIF (fasted state simulated intestinal fluid; pH = 6.5) and FeSSIF (fed state simulated intestinal fluid; pH = 5) were applied for the present work. Microsphere samples (20 mg) were incubated in 1.5-mL microcentrifuge tubes containing 1 mL medium and allowed to be shaken at 75 rpm and 37°C using an orbital incubator (SI 150, Stuart Scientific Co., UK). The suspensions were centrifuged at 10000 rpm for 10 min by a microcentrifuge (5415C, Eppendorf Co., Germany). The precipitate and supernatant of each sample were then separated and analyzed. Microspheres integrity was studied by light and scanning electron microscope. An enzymatic assay kit was applied for the quantification of L-lactic acid (L-LA) as the product of microspheres degradation using the method described previously (17). The amount of protein released was determined in the supernatants, using the above-mentioned fluorimetric assay. SDS-PAGE electrophoresis method was also applied for the study of physical stability of the trapped protein in microspheres following the exposure to GIT media.

## Results and Discussion

The present study was undertaken to evaluate the *in-vitro *stability of protein-loaded polyester microspheres in GI tract. Since the hypothesis and rational were the uptake of intact microspheres, the ideal situation was the preparation of particles with the least release during the stay in the lumen. Meanwhile, the maintenance of the protein stability is one of the major criteria for the successful design of a protein delivery system. 

Following our previous research on the stability of various polyesters in simulated GI fluids, Resomer® RG505, RG756 and R207 were selected for the preparation of microspheres for oral protein delivery. rhGH-loaded microspheres were fabricated by stabilizing and encapsulating the protein by two double emulsion methods, as mentioned earlier. The studied formulations and their methods of preparation and characteristics are summarized in Table 1. 

**Table 1:** Characteristics and method of the preparation of various rhGH-loaded microsphere formulations (n = 3).

**Formulation**	**Resomer** ^®^ ** polymer**	Fabrication method*	**Residence time in hardening bath (h)**	**Physiogel addition**	**Zn:rhGH ratio**	**% Loading**	**% Release in buffer after 24 h**
A	RG756	I	7	-	-	24.94	74.86
B	RG756	I	18	-	-	23.02	60.43
C	RG756	II	7	-	100	29.13	Not determined
D	RG756	II	18	-	100	30.12	16.10
E	RG756	I	18	+	-	42.07	Not determined
F	RG756	II	18	+	40	51.90	32.21
G	RG756	II	18	+	100	57.04	25.06
H	RG505	II	18	+	100	63.52	42.06
I	R207	II	18	+	100	49.98	21.78

*: Method I is a usual double emulsion technique and method II is a modified one by which a protein is micronized through complexation with zinc, followed by microencapsulation

**Figure 1:**
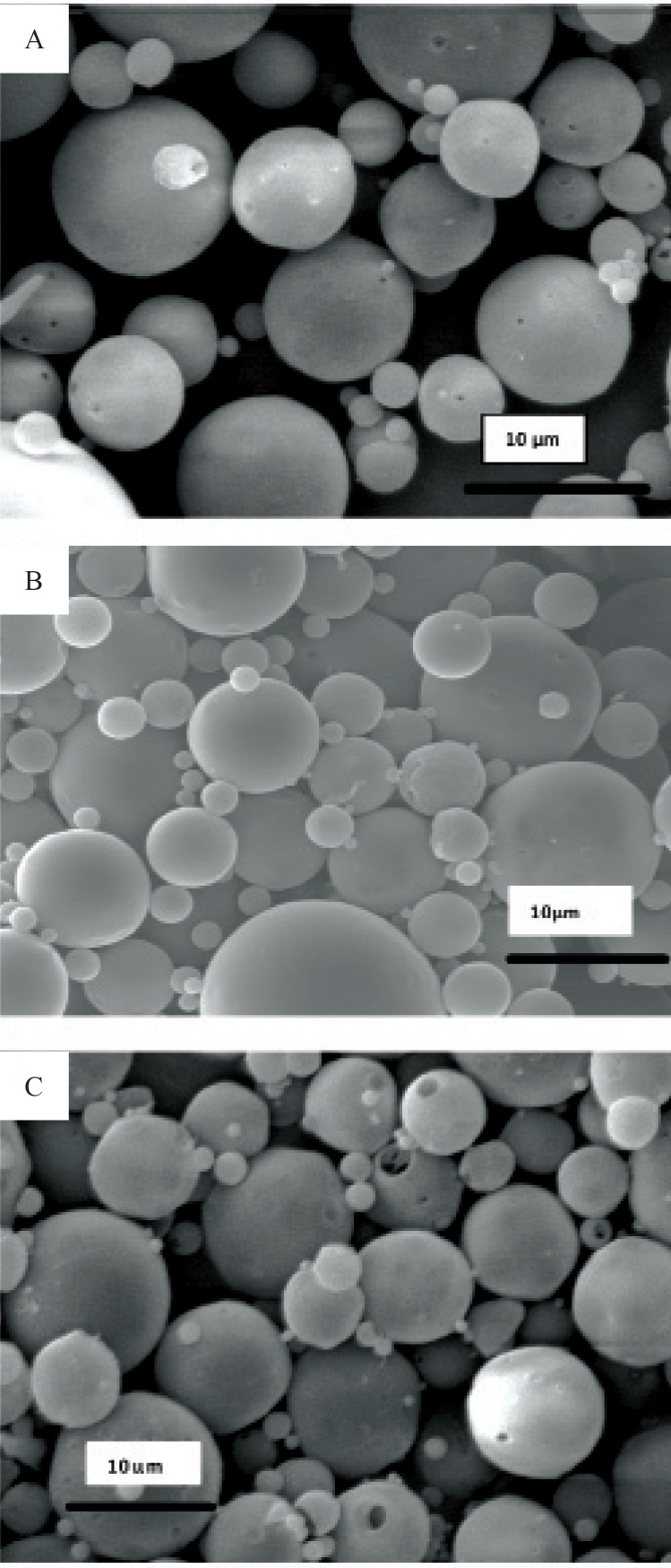
Scanning electron micrograph of microspheres prepared. a) formulation G, b) formulation H, c) formulation I

Obtained results showed no significant influence of the residence time in the hardening bath on the amount of protein loading in microspheres. However, shorter times were resulted to more initial release of protein which could be attributed to incomplete solvent evaporation and therefore, higher surface porosity. Microspheres obtained by shaking in PVA solution for 18 h possessed regular and spherical shapes with smooth surfaces and a few pores as visualized by SEM (Figure 1). 

Literature review has shown the evidence for the GI absorption of micronized particles by various size-dependent mechanisms, but it is noticeable that there are controversies on the optimum size of absorbable particles. It is generally accepted that smaller particles show better uptake than larger ones (21-24). The size may also play an important role in the fate of absorbed microparticles (25). In this work, SEM studies showed that the prepared microspheres were mainly below 10 μm in diameter. This result is consistent with the size range cutoff reported for transcellular uptake of particles (26). 

Microencapsulation process includes several detrimental steps that influence the protein stability through its exposure to aqueous/organic interface or induction of shear stress or heat during homogenization. In method II, PLGA microspheres were made by precipitating rhGH from solution, following the addition of zinc solution and then encapsulating the Zn-protein complex. It has been shown that the precipitate would contain micronized protein particles (18). This method was used in this study as an alternative in order to reduce the protein/solvent interface without the need for special instruments or large amounts of protein (19). In addition, there is significant evidence of stabilization effects of zinc on proteins and even it is believed that growth hormone is naturally stored as a reversible zinc complex in the secretary granules of the anterior pituitary (27). On the other hand, it has been reported that zinc ions can interact with the free carboxyl groups at the PLGA molecules, leading to both the alteration of drug release profile and the increase of glass transition temperature of polymer (28).

**Figure 2:**
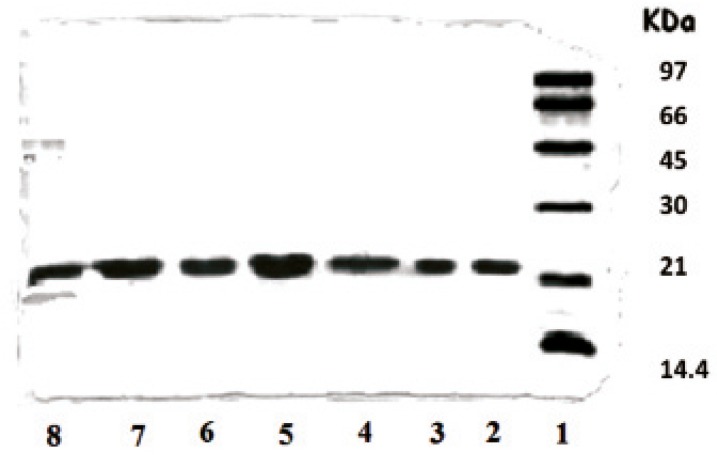
SDS-PAGE results of rhGH encapsulated in various microspheres. Lane 1: Standard protein markers, Lane 2: Standard rhGH, Lane 3: Standard rhGH complexed with zinc (1:100 molar ratio), Lanes 4-7: F, G, H and I microspheres, respectively, fabricated by method II, Lane 8: E microspheres fabricated by method I

The effect of microencapsulation on rhGH stability in the presence and absence of the zinc additive was evaluated by SDS-PAGE technique in order to detect any protein aggregation or fragmentation reactions which might occur within microspheres (29, 30). Figure 2 shows the results of electrophoresis analysis obtained by direct loading of microspheres in the wells. As depicted, rhGH encapsulated through method I (in the absence of zinc) exhibited physical instability (lane 8). 

Comparison of lanes 2 and 3 shows that the complexation with zinc is a reversible phenomenon and the protein can convert to its monomer during the process. No covalently aggregated or fragmented protein was detectable in microspheres loaded with Zn-rhGH complex (lanes 4-7). As presented in Table 1, the presence of zinc in formulations also led to a lower initial release of protein. Therefore, it was concluded that method II was less deleterious for protein physical stability and a better choice for our research on rhGH microencapsulation.

Higher protein entrapment was achieved by the addition of Physiogel^®^ into the inner aqueous phase. Physiogel^®^ is a succinylated gelatin used as plasma substitutes in human and can be used as a co-encapsulated additive without any safety considerations. Both PVA and Physiogel^®^ molecules act as surface active agents, capable of protecting proteins from shaking/shearing-induced aggregation, by competitively accumulating with proteins at hydrophobic surfaces/interfaces and/or by direct binding to proteins. In addition, the viscosity increase induced by PVA and Physiogel^® ^may also be useful for restraining the motion of protein backbone and inhibiting its aggregation. Furthermore, it is reported that gelatin is able to protect proteins from heat-induced aggregation *e.g. *during homogenization (30).

**Figure 3:**
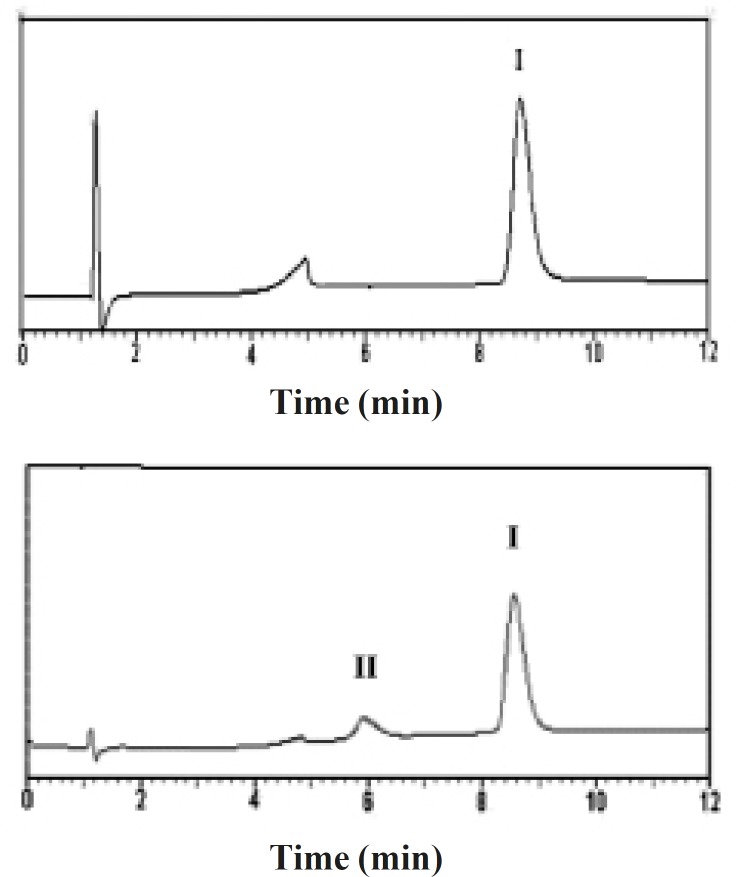
Reversed-phase HPLC results of (*a*) standard solution of rhGH, and (*b*) rhGH extracted from G microspheres (peak I: native rhGH, peak II: Physiogel^®^).

Developed reversed-phase chromatography analysis was performed for the assessment of chemical stability of the entrapped rhGH and separation of its deamidated and oxidized forms (20). Figure 3 depicts the reversed-phase HPLC results of a standard rhGH solution and rhGH extracted from G microspheres (Table 1), indicating that the encapsulation process caused no detectable change in the protein chemical stability.

Three microsphere formulations (G, H and I), differed only in polymer composition, were used in this part of the study. These microspheres were selected based on the lower *in-vitro *initial release and higher protein loading (Table 1) and therefore, their stability was studied in various simulated GI media. It should be noted that the incubation periods applied in these experiments were much longer than the reported residence times of such particles in GI tract.

Lactate assay was applied as a fast, sensitive and specific method for the detection of polymer degradation. The method validation was performed in our lab and no interfere with the media constitutes was observed. The obtained results are presented in Table 2. 

**Table 2:** Degradation percent of protein-loaded microspheres following the incubation for various times, calculated on the basis of free lactate production (n = 3).

**Formulation code**	**Resomer** ^®^	**Incubation medium (incubation time)**			
		**SGF-a** **(6 h)**	**SIF-a** **(12 h)**	**FaSSIF** **(12 h)**	**FeSSIF** **(12 h)**
**G**	RG756	0.326	1.6	0.286	0.225
**H**	RG505	0.433	7.7	0.420	0.414
**I**	R207	0.327	0.52	0.204	0.188

H microspheres did not show proper stability in simulated GI fluids and especially in SIF-a. Generally, all rhGH-loaded microspheres were more sensitive than their related blank samples to the harsh conditions of the incubation media. This could be due to the differences occurred in the surface or internal structure of microspheres by adding the protein or other additives to the formulation. As it was shown for blank microspheres, RG756 and R207-made microspheres (G and I) were recognized as much better carriers than RG505 for tailoring protein formulations that could be delivered orally.

Release percents of rhGH from G and I microspheres following the incubation in simulated GI fluids are shown in Table 3. 

**Table 3:** Release percent of rhGH from G and I microspheres following the incubation in simulated GI fluids (n = 3).

**Medium (Incubation time)**	**Release percent of rhGH from microspheres**	
	**Formulation I**	**Formulation G**
SGF-b (6 h)	8.23	11.20
SIF-b (12 h)	13.43	14.91
FaSSIF (12 h)	8.42	10.56
FeSSIF (12 h)	7.98	9.43
SGF-b (3h) + SIF-b (6 h)	17.84	19.37

*For prevention of any interfere in protein assay, the media without enzymes had been selected for these experiments

The percent of released rhGH from microspheres after incubation in both simulated stomach and intestine fluids for 6 and 12 h, respectively, was totally less than 20%, that was acceptable considering the initial release from microspheres in phosphate buffer saline (pH = 7.4), shown in Table 1.

**Figure 4:**
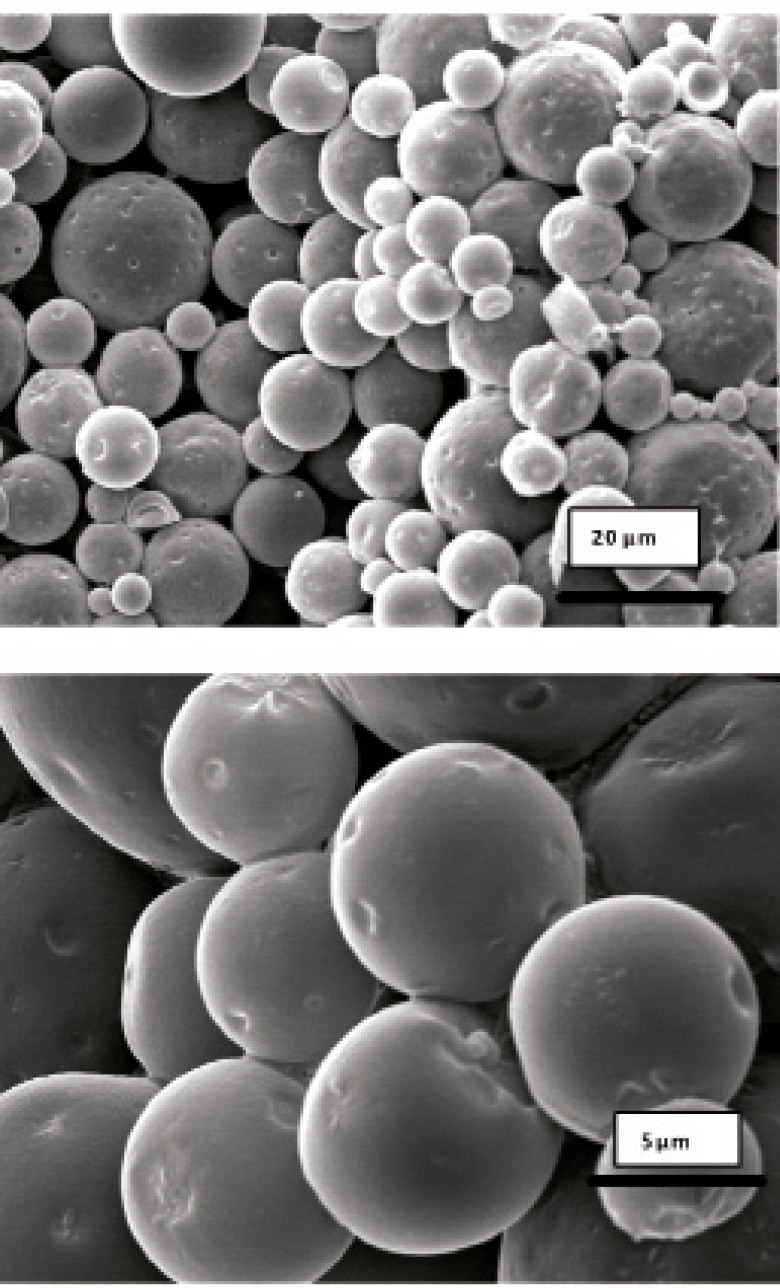
Scanning electron micrographs of G microspheres after incubation at 37°C in SGF-a for 6 h.

**Figure 5:**
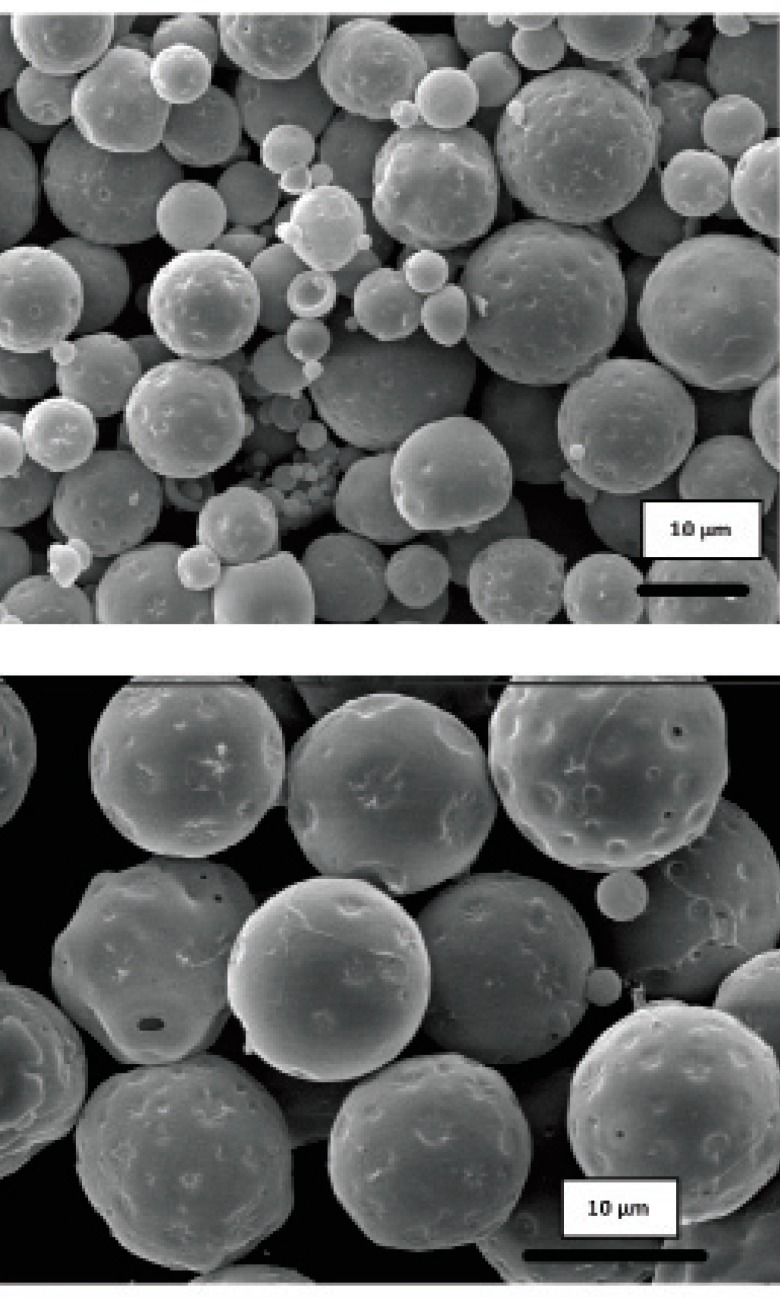
Scanning electron micrographs of G microspheres after incubation at 37°C in SIF-a for 12 h.

**Figure 6:**
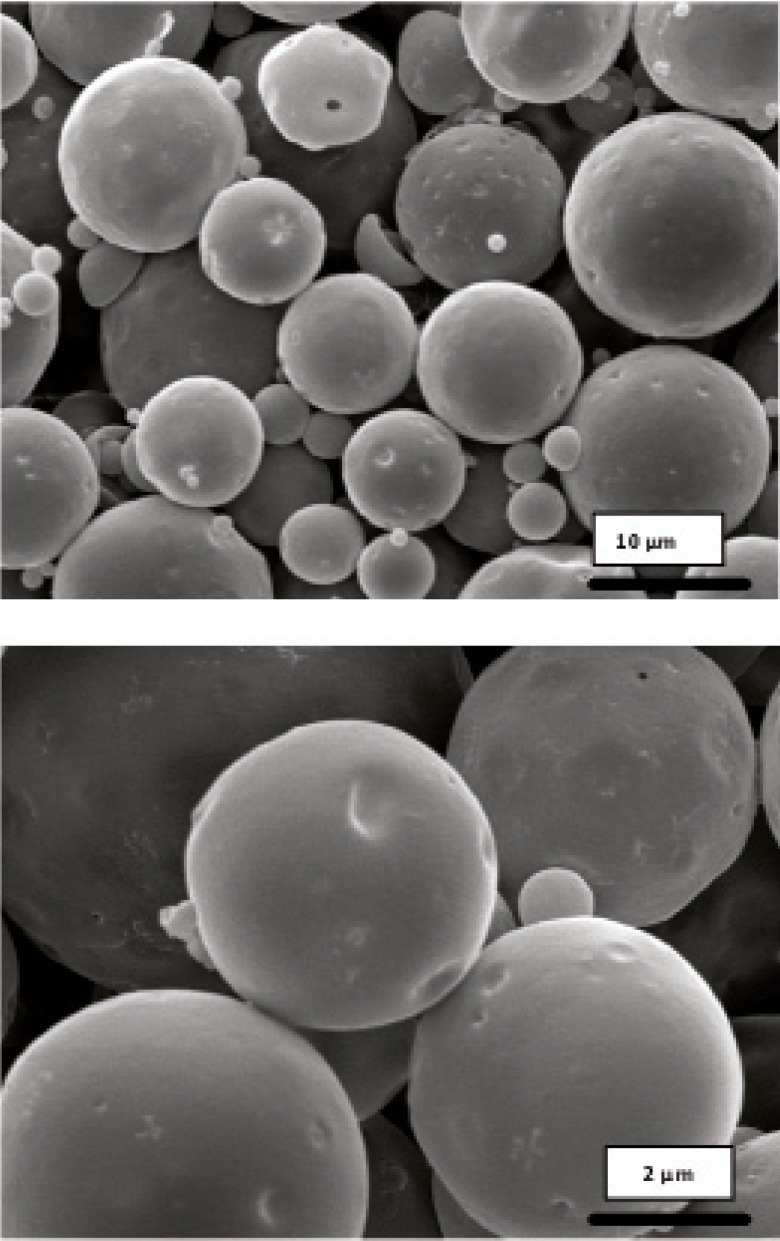
Scanning electron micrographs of G microspheres after incubation for 12 h at 37°C in FaSSIF.

**Figure 7:**
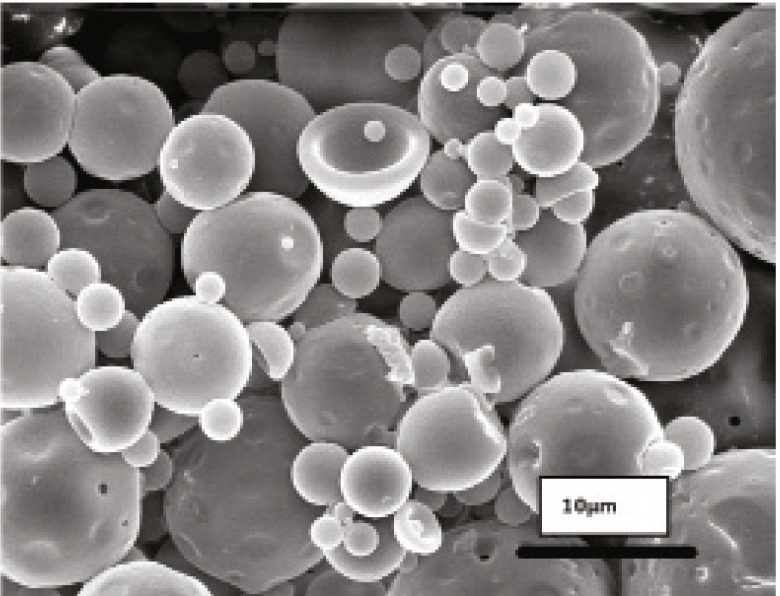
Scanning electron micrographs of G microspheres after incubation for 12 h at 37°C in FaSSIF.

**Figure 8:**
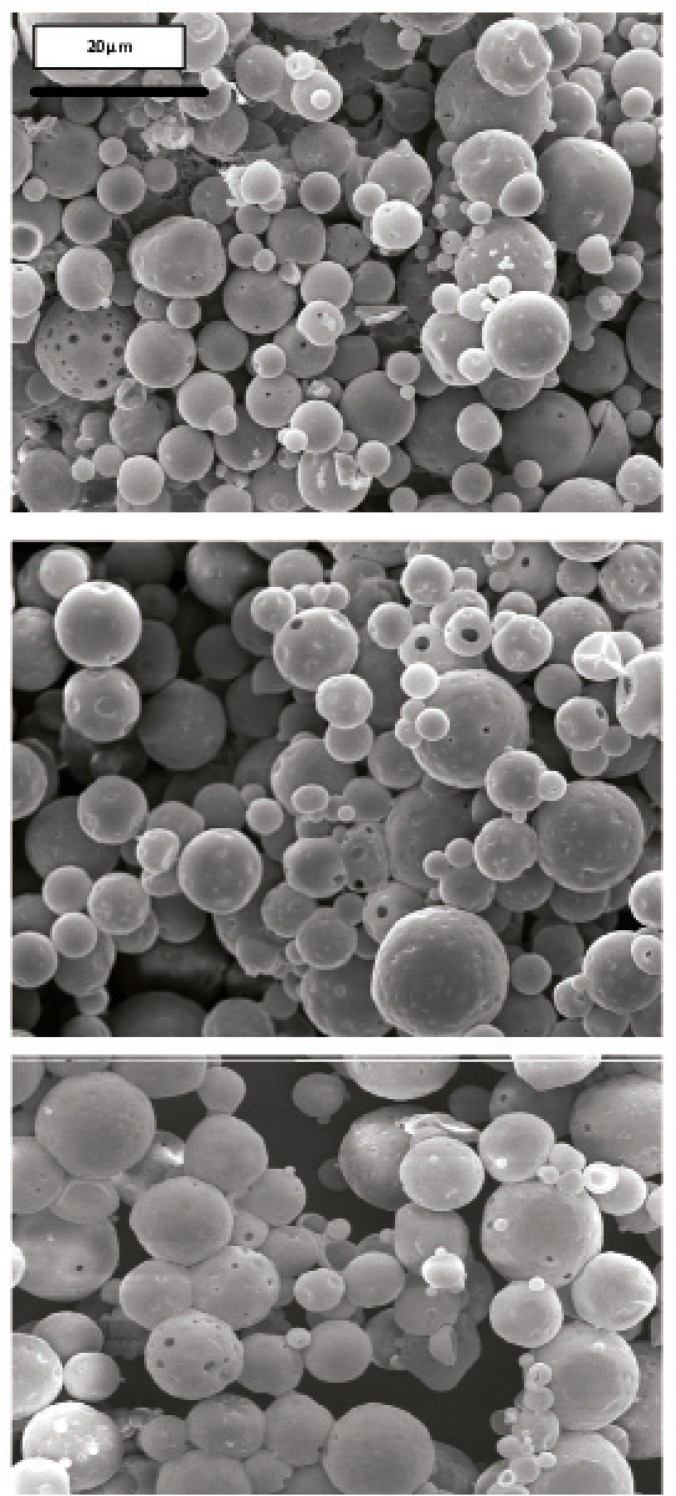
Scanning electron micrograph of G microspheres after incubation for 3 h at 37°C in SGF-a followed by a 6-hr incubation at 37°C in SIF-a

Morphology and degradation of microspheres were also studied using the scanning electron microscopy. Figures 4, 5, 6, 7, 8 are typical scanning electron micrograph of G microspheres incubated for various times in a different media. The size of microspheres did not significantly change following the contact with this different media. Surfaces of microspheres appeared smooth but more pores were visible following the incubation mostly in SIF-a (Figure 5). Electrophoresis analysis revealed that the remained rhGH within microspheres maintained its structural integrity during the incubation and therefore, irreversible aggregation was not occurred. 

## Conclusion

Biodegradable microspheres loaded with rhGH can be successfully prepared from PLA/PLGA polymers by the double emulsion method. The *in-vitro *release study in various simulated GI fluids exhibited a low release over an extended period, while the stability analysis revealed that the encapsulated rhGH within microspheres maintained its structural integrity during the incubation. This study shows that Resomer^®^ R207 and RG756 microspheres could be considered as promising systems for oral delivery of pharmaceutical proteins.
